# Cerebrovascular manifestations and alteration of coagulation profile in scorpion sting: A case series

**DOI:** 10.4103/0972-5229.40944

**Published:** 2008

**Authors:** Suman Sarkar, Prithwis Bhattacharya, Anil Paswan

**Affiliations:** **From:** Department of Anesthesiology, IMS Banaras Hindu University, Varanasi, Uttar Pradesh, India

**Keywords:** Disseminated intravascular coagulation, scorpion sting, stroke

## Abstract

Cerebrovascular manifestations are uncommon presentations of scorpion sting in the Indian subcontinent. A prospective study was carried out on 42 patients with scorpion sting in the intensive care unit (ICU) of the Institute of Medical Sciences, Banaras Hindu University, Varanasi-05, INDIA, during the period of May 2005 to October 2007. In all the patients detailed history, physical examination with a specific neurological examination and routine biochemical testing and fundus examination were done. Computerized tomography and magnetic resonance imaging were done in cases with neurological deficit. All these patients also underwent a complete hematological, rheumatologic and cardiovascular work-up for stroke. Cerebrovascular involvement was noted in three patients (7.15%). Hemorrhagic stroke was noted in two patients (4.77%) and thrombotic stroke was noted in one patient (2.39%). The mean time of presentation of neurological symptoms was 3 days. Contrary to world literature, there have been no reports of cranial nerve palsies or neuromuscular involvement in our series.

## Introduction

Scorpion sting is an acute life-threatening, time-limiting medical emergency more commonly seen in villagers.[1] Among the 86 species of scorpions in India, Mesobuthus tamulus and Palamneus are of medical importance.[2] Cardiovascular effects are particularly prominent following the stings by Indian red scorpion (Mesobuthus tamulus).[3] Cerbrovascular manifestations are uncommon presentations of scorpion sting in the Indian subcontinent.

During the period of May 2005 to October 2007, a prospective study was done in patients with scorpion sting admitted to the intensive care unit of the Institute of Medical Sciences, Banaras Hindu University, Varanasi-05, INDIA. The diagnosis was based on positive history of scorpion sting, with scorpion being seen or killed by relatives or bystanders.

In all the patients history, physical examination with a specific neurological examination and routine biochemical testing and fundus examination to specifically look for changes in the retinal vessels due to longstanding hypertension were done. Vitals were recorded on arrival and thereafter at one hourly interval. Electrocardiogram (ECG) was recorded to detect any evidence of scorpion sting induced myocarditis and to detect any evidence of left ventricular hypertrophy due to longstanding hypertension.

Cases with neurological deficit were included in the further work-up. Computerized tomography and magnetic resonance imaging were done in cases with neurological deficit. All these patients also underwent a complete hematological work up in the form of complete blood count, hematocrit, peripheral smear, platelet count, coagulation profile, fibrin degradation products, serum homocysteine, rheumatological work-up of antinuclear antibody and cardiovascular work-up of lipid profile, transthoracic echocardiogram and carotid doppler. All these investigations were done at the time of admission.

Out of the total of 42 patients with documented scorpion sting, focal neurological deficit was noted in three patients (7.15%). The commonest presentation of neurological deficit was hemiparesis, observed in all three cases. All the three patients were males. Their mean age was 35 years (range from 18 to 49 years). Hemorrhagic stroke was noted in two patients (4.77%) and thrombotic stroke was noted in one patient (2.39%). Among the patients with hemorrhagic stroke, one patient had an intraventricular hemorrhage, while one patient had hemorrhage in the putamen. The patient with thrombotic stroke had involvement of the middle cerebral artery (MCA) territory - due to disseminated intravascular coagulation (DIC). Patient with DIC producing a thrombotic stroke presented at 3 days after the scorpion sting, while patients with hemorrhagic stroke presented within 48 h of the scorpion sting. Patient with thrombotic stroke due to DIC had documented defibrination. All the three patients had a normal carotid doppler and a normal transthoracic echocardiogram with no evidence of left ventricular hypertrophy. All the patients with stroke had no evidence of myocarditis or pulmonary edema on clinical examination or echocardiogram. One patient with intralobar hemorrhage died on the fifth day of sting due to raised intracranial tension and coning. The other two patients did exceedingly well and recovered within a period of 3 months.

Prazosin was initiated in all the three patients with scorpion sting. Patients with stroke, both thrombotic and hemorrhagic, were managed conservatively with prazosin and supportive measures.

## Discussion

Scorpion venoms are species-specific complex mixtures of short neurotoxic proteins.[4] Alpha-receptor stimulation by the toxin results in hypertension, tachycardia, myocardial dysfunction, pulmonary edema.[5] Raised angiotensin I levels have also been documented, which facilitates the sympathetic outflow through conversion to angiotensin II.[6] The unopposed effects of alpha-receptor stimulation lead to myocarditis. Acute rise in blood pressure due to sympathetic stimulation, rupture of unprotected perforating arteries, intracerebral hemorrhage and cerebral infarction due to DIC and central respiratory failure are reported in scorpion stings.[7–9]

Our two patients with hemorrhagic stroke had an acute rise in blood pressure - of around 260/130 mm of Hg - at the time of admission due to autonomic storm, which would explain the hemorrhage inside the brain. By the use of carotid doppler, echocardiogram, electrocardiogram, lipid profile, we excluded patients with longstanding hypertension, which would have caused stroke in these patients. The other patient with thrombotic stroke due to DIC could be explained based on the toxin-induced alteration in the coagulation system inducing a defibrination syndrome.[7] There have been reports of cerebral vasospasm induced infarcts in the brain. Thacker *et al.* reported a case of scorpion sting induced multiple cerebral infarcts with optic neuropathy.[10]

There have been a number of limitations in our study as vasospasm could not be proved by angiography. Protein C, Protein S and antithrombin III estimation was not done in thrombotic stroke in view of the fact that the deficiency of Protein C, Protein S and anti thrombin III usually produce venous infarcts rather than arterial infarcts, as in our series. Transesophageal echocardiogram was not done in our cases, which would have picked up thrombus in the heart missed by transthoracic echocardiogram. But it would be a highly unlikely event to expect thrombus in a patient with a well-contracting myocardium with no evidence of hypertrophy or dilatation of heart chambers.

## Conclusion

Cerebrovascular manifestations were seen in three (7.15%) patients. Treatment with prazosin, if initiated early, may prevent many cerebrovascular manifestations of scorpion sting.

## References

[1] Bawaskar HS, Bawaskar PH (1989). Sting by red scorpion (Buthus tamulus) in Maharashtra State, India: A clinical study. Trans Roy Soc Med Hyg.

[2] Erfati P, Bettini S (1978). Epidemiology, symptomatology and treatment of buthinae stings. Arthopod Venoms. Handbook of Experimental Pharmaco-logy.

[3] Bawaskar HS, Bawaskar PH (1998). Indian red scorpion envenoming. Indian J Pediatr.

[4] Zlotkin E, Miranda F, Lissitzky S (1972). Proteins in scorpion venoms toxic to mammals and insects. Toxicon.

[5] Bawaskar HS, Bawaskar PH (1992). Management of cardiovascular manifestations of poisoning by the Indian red scorpion (Mesobuthus tamulus). Br Heart J.

[6] Gueron M, Adolph RJ, Grupp IL, Gabel M, Grupp G, Fowler NO (1980). Hemo-dynamic and myocardial consequences of scorpion venom. Am J Cardiol.

[7] Kochar DK, Singh P, Sharma BV, Saini G, Aggarwal P, Gauri LA (2002). Scorpion envenomation causing hemiparesis. J Assoc Physic India.

[8] Tiwari SK, Gupta GB, Gupta SR, Mishra SN, Pradhan PK (1988). Fatal stroke following scorpion bite. J Assoc Physics India.

[9] Rai M, Shukla RC, Varma DN, Bajpai HS, Gupta SK (1990). Intracerebral hemorrhage following scorpion bite. Neurology.

[10] Thacker AK, Lal R, Misra M (2002). Scorpion bite and multiple cerebral infarcts. Neurol India.

